# Bladder metastasis from gastric cancer presenting as acute post-renal obstruction during immunotherapy: a case report

**DOI:** 10.3389/fonc.2026.1848708

**Published:** 2026-06-30

**Authors:** Jiaojiao Hou, Xiaolei Yin, Lili Mi, Xin Han, Ning Li, Fei Yin

**Affiliations:** Department of Gastroenterology, the Fourth Hospital of Hebei Medical University, Shijiazhuang, Hebei, China

**Keywords:** acute kidney injury, bladder metastasis, case report, gastric cancer, immunotherapy

## Abstract

Bladder metastasis from gastric cancer is extremely rare and may be misdiagnosed, particularly during immunotherapy, when new urinary symptoms can be attributed to genitourinary immune-related adverse events. We report a case of a 35-year-old man with advanced gastric cancer who initially responded to chemotherapy combined with sintilimab, an anti–Programmed Death-1 (PD-1) inhibitor. After four cycles, he developed acute kidney injury and progressive obstructive uropathy, including bilateral hydronephrosis and diffuse irregular bladder wall thickening, whereas the primary gastric lesion remained stable. Routine urinalysis revealed no such abnormalities. Cystoscopy revealed patchy follicular mucosal elevations with non-visualization of both ureteral orifices. Ureteral stent placement failed, and bilateral percutaneous nephrostomy was performed, resulting in partial recovery of the renal function. Bladder biopsy confirmed a metastatic poorly differentiated adenocarcinoma with signet-ring cell features. Immunohistochemistry was positive for caudal-type homeobox 2 (CDX2), cytokeratin 20 (CK20) and Villin, consistent with gastric origin. Despite urinary diversion, the disease progressed rapidly, leading to multiple organ failure and death in the patient. This case highlights the importance of early cystoscopy and targeted biopsy in patients receiving immunotherapy who develop new urinary symptoms or atypical urinary tract imaging findings to distinguish metastatic disease from immunotherapy-related toxicity.

## Introduction

Gastric cancer is a leading cause of cancer-related mortality worldwide. Common metastatic sites include the liver, peritoneum, and lymph nodes, whereas bladder metastasis is exceedingly rare (1, 2). Such atypical metastases are often associated with aggressive tumor biology and poor prognosis. Immune checkpoint inhibitors combined with chemotherapy are the standard first-line treatment for advanced gastric cancer (3). However, atypical response patterns, including hyperprogression and discordant responses, have been increasingly recognized and may complicate clinical evaluations (4, 5).

Notably, new-onset lower urinary tract symptoms during immune checkpoint inhibitor–based therapy are frequently attributed to genitourinary immune-related adverse events. However, immune-related cystitis or ureteritis may closely mimic malignant involvement, particularly when imaging shows bladder wall thickening and hydronephrosis (6, 7). In such settings, distinguishing immunotherapy-related toxicity from metastatic progression is critical because delayed diagnosis may result in irreversible renal damage and missed opportunities for timely oncologic management.

Here, we report a rare case of bladder metastasis presenting as acute post-renal obstruction after an initial response to immunochemotherapy. This case highlights the important diagnostic pitfalls and clinical considerations associated with the use of immunotherapy.

## Case description

A 35-year-old male driver without notable previous medical history was admitted in January 2025 due to one month of upper abdominal pain and melena, along with progressive weight loss. The patient denied experiencing hematemesis, abdominal distension, nausea, vomiting, regurgitation, acid reflux or heartburn. He also reported progressive fatigue and a decline in physical strength since the onset of symptoms. He denied smoking, alcohol abuse, and long-term medication use, with no history of radiation exposure or contact with toxic substances. His family history was remarkable for lung cancer in his maternal grandfather and liver cancer in his maternal uncle. His mother had heart disease, and his father was healthy. On physical examination, the patient was pale and presented with mild epigastric tenderness. No rebound tenderness or muscle guarding was observed. Shifting dullness was negative, and no other significant abnormalities were observed.

Laboratory tests showed hemoglobin of 74 g/L, Carcinoembryonic antigen (CEA) of 7.0 ng/mL, and Carbohydrate antigen 19-9 (CA19-9) levels of 661 U/mL. Esophagogastroduodenoscopy revealed an ulcerative infiltrative lesion extending from the gastric body to the antrum, consistent with Borrmann type-IV gastric cancer. Histopathological examination confirmed poorly differentiated adenocarcinoma. Contrast-enhanced computed tomography (CT) revealed diffuse gastric wall thickening and multiple lymph node metastases. The clinical stage was cT4N2M1 (stage IV). Molecular analysis identified a tumor protein p53 (TP53) mutation, microsatellite stability, low tumor mutational burden, PD-L1 Combined positive score (CPS) of 5, and fibroblast growth factor receptor 2 (FGFR2) amplification.

**Figure 1:**
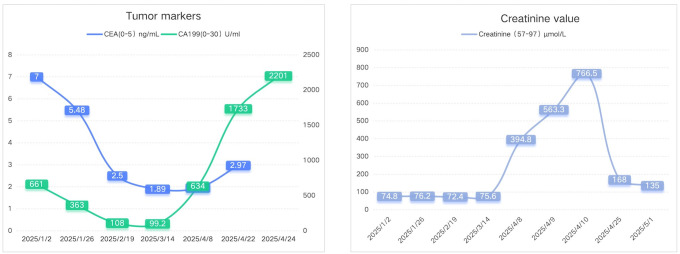
Dynamic changes in serum tumor markers (CEA and CA19-9) and creatinine levels during treatment.

**Figure 2:**
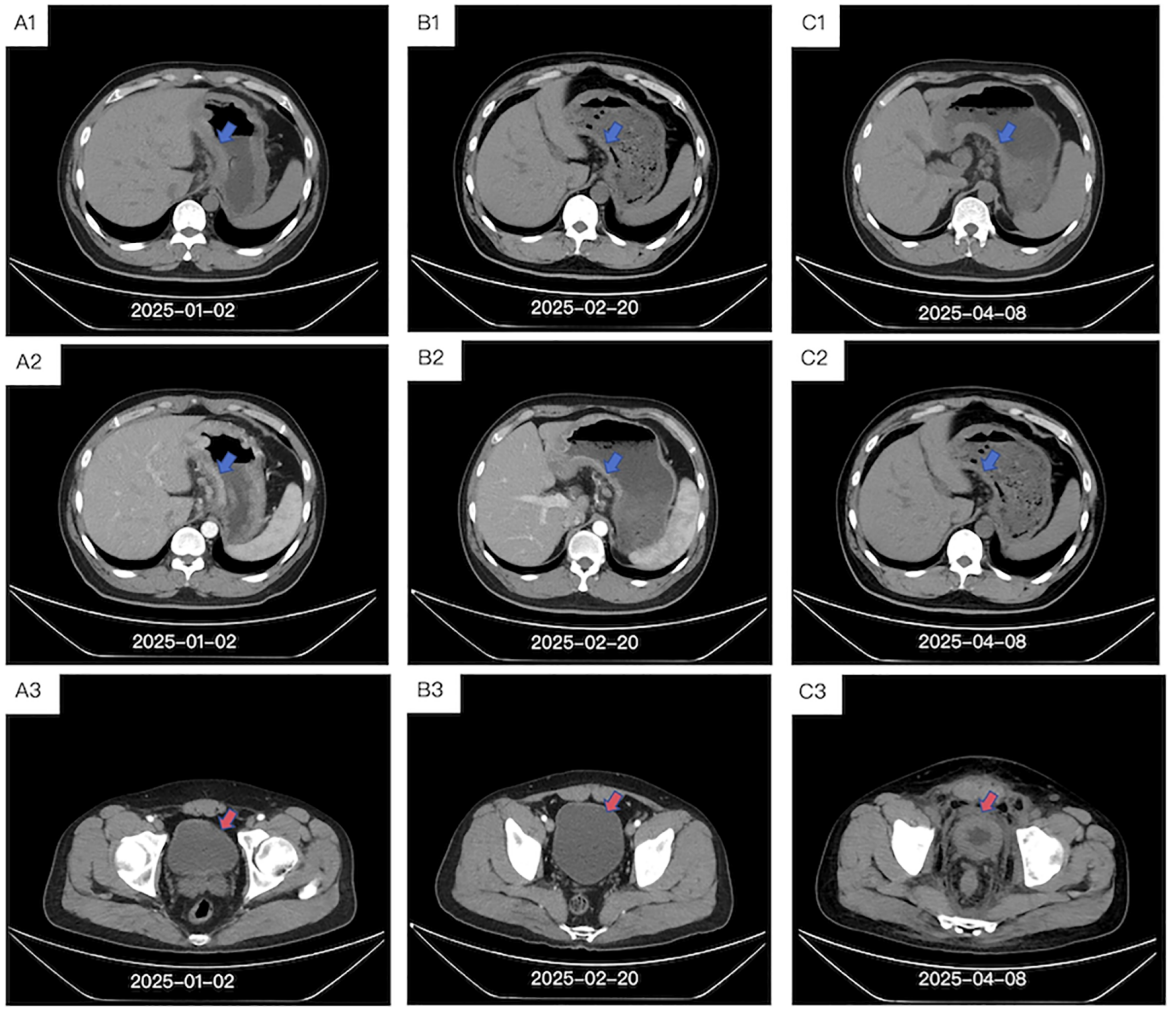
Computed tomography (CT) images during the disease course. **(A1, A2)** Baseline CT (January 2, 2025) showing diffuse gastric wall thickening (blue arrows): **(A1)** non-contrast scan; **(A2)** contrast-enhanced scan. **(A3)** No bladder wall thickening (red arrow). **(B1, B2)** After two cycles of treatment (February 20, 2025) demonstrating reduced gastric wall thickening (blue arrows): **(B1)** non-contrast scan; **(B2)** contrast-enhanced scan. **(B3)** No bladder wall thickening (red arrow). **(C1, C2)** At disease progression (April 8, 2025), both **(C1)** and **(C2)** are non-contrast scans due to impaired renal function: the gastric lesion shows no significant progression (blue arrows). **(C3)** Irregular bladder wall thickening consistent with metastatic involvement (red arrow).

The patient received the capecitabine plus oxaliplatin (XELOX) regimen (oxaliplatin 130 mg/m² on day 1, capecitabine 1000 mg/m² twice daily on days 1–14) plus sintilimab 200 mg on day 1, in 21-day cycles. After two cycles, the tumor markers decreased markedly, and imaging assessment demonstrated a partial response (PR) (**Figures 1**; **2**).

Upon completion of the fourth treatment cycle, the patient developed urinary incontinence without dysuria or hematuria, along with progressive deterioration of renal function. His serum creatinine level increased to 394.8 μmol/L and continued to rise to 766.5 μmol/L within 48 hours (**Figure 1**). Routine urinalysis revealed no obvious abnormalities. CT imaging revealed bilateral hydronephrosis, diffuse irregular bladder wall thickening, and mild peritoneal invasion, while the primary gastric lesion remained stable (**Figure 2**). In view of the rapid deterioration of renal function, urinary tract ultrasonography was performed, which detected bilateral separation of the renal collecting system. Subsequent cystoscopy revealed patchy follicular protrusions predominantly located in the bladder trigone and posterior wall, with invisible bilateral ureteral orifices. Ureteral stent implantation failed, and bilateral percutaneous nephrostomy was performed, leading to partial recovery of renal function after surgery.

**Figure 3:**
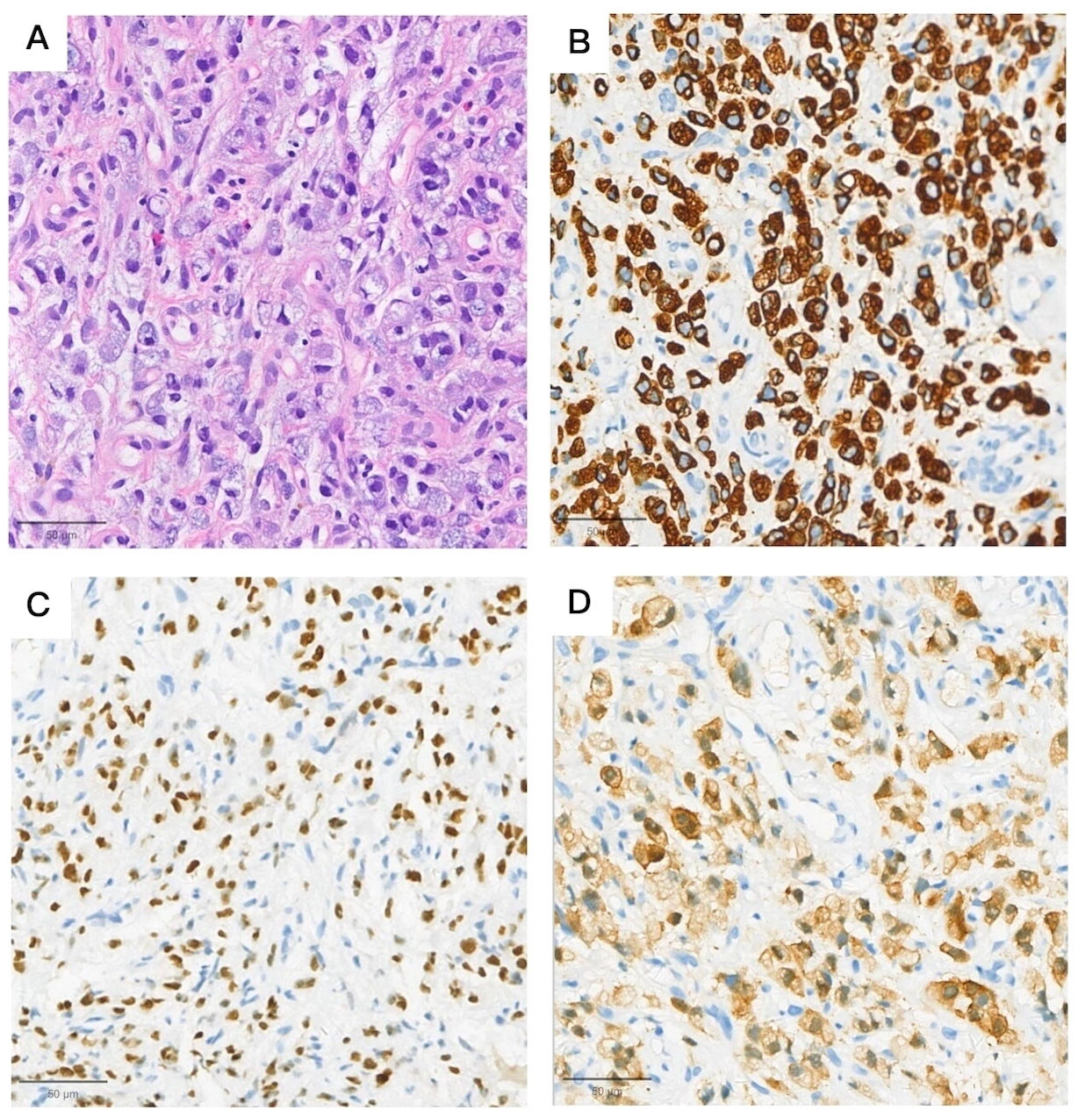
Histopathological and immunohistochemical findings of the bladder lesion. **(A)** Hematoxylin and eosin staining showing poorly differentiated adenocarcinoma with signet-ring cell features. **(B)** Tumor cells showing strong membranous and cytoplasmic CK20 positivity. **(C)** Nuclear positivity for CDX2 in the tumor cells. **(D)** Positive staining for Villin, supporting gastrointestinal origin. Scale bars = 50um.

Histopathological examination of the bladder biopsy revealed poorly differentiated adenocarcinoma with signet-ring cell (SRC) features on hematoxylin and eosin staining. Immunohistochemistry revealed positivity for CK20, CDX2, and Villin, supporting a gastrointestinal origin (**Figure 3**). Given the patient’s known primary gastric carcinoma and histomorphological concordance, the bladder lesion was interpreted as a metastatic gastric adenocarcinoma.

**Figure 4:**
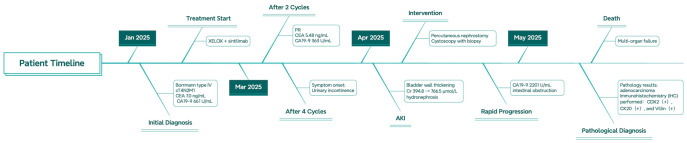
Clinical timeline depicting the disease course and treatment responses. PR, partial response; AKI, acute kidney injury; IHC, immunohistochemistry; Cr, serum creatinine.

Despite these interventions, the tumor markers increased significantly, and the patient developed intestinal obstruction, necessitating emergency surgical treatment. Nevertheless, his clinical condition deteriorated rapidly, and he succumbed to multiple organ failures in late May 2025. The timeline of the major clinical events is shown in **Figure 4**.

## Discussion

Bladder metastasis from gastric cancer is exceedingly rare (1, 2), particularly in young patients. The mechanisms remain unclear, but several interacting factors are likely to be involved. First, the histological subtype may provide a critical biological basis: SRC histology, reported in over 75% of cases (1), is driven by Cadherin 1 (CDH1) mutations and loss of E-cadherin function, which promotes cellular dissociation, diffuse infiltration, and early serosal spread, thereby facilitating hematogenous and lymphatic dissemination (8, 9). Second, the “seed and soil” hypothesis may explain bladder tropism, as the richly vascularized and lymphatic microenvironment of the bladder can support tumor colonization (10, 11). Third, the metastatic cascade may be further amplified by epithelial-mesenchymal transition (EMT) and tumor-derived exosomes, which enhance tumor cell mobility and remodel the distant stromal/immune landscape to establish a pre-metastatic niche (12, 13). Finally, successful colonization requires tumor cells to overcome barriers such as detachment-induced apoptosis and immune surveillance; thus, only subclones with appropriate molecular programs form secondary lesions. Beyond CDH1, alterations in E74-like ETS transcription factor 3 (ELF3) and polymeric immunoglobulin receptor (PIGR) have also been implicated in unusual metastatic patterns (14–18). Together, these mechanisms suggest that bladder metastasis, although rare, is biologically plausible; however, the specific mechanisms require further investigation.

A search of the Public MEDLINE (PubMed) database was performed to identify peer-reviewed, full-text case reports published in English since 1984 that described gastric cancer with bladder metastasis in detail. To date, 26 case reports (excluding the present case) encompassing 32 patients have documented secondary bladder metastasis arising from gastric tumors, underscoring its rarity. Compared with previously reported cases of bladder metastasis from gastric cancer, the present case shows overlapping clinicopathological and radiological features, including signet-ring cell carcinoma and diffuse bladder wall thickening with associated hydronephrosis. However, several distinctive aspects warrant further attention. First, urinary incontinence was the main presenting symptom in our patient, whereas hematuria was the predominant manifestation in the literature (e.g., 12 of 18 patients in a pooled analysis) (2). We speculate that incontinence resulted from diffuse tumor infiltration of the bladder neck and sphincter region, leading to impaired urethral closure. This non-hematuric presentation has rarely been emphasized in previous reports (19–21). Second, bladder metastasis can develop during immunochemotherapy. Importantly, the primary gastric lesion exhibited a partial response, whereas bladder involvement progressed discordantly, which differs from most earlier reports in which bladder metastases occurred during conventional chemotherapy alone or after a long post-gastrectomy interval (22, 23). Collectively, these findings suggest that clinicians should consider bladder metastasis in patients receiving immunotherapy who present with non-hematuric lower urinary tract symptoms, even in the absence of hematuria.

This case also illustrates a discordant response pattern in which the primary gastric lesion remained stable, while new metastatic disease rapidly emerged. These patterns may reflect the underlying tumor heterogeneity, clonal selection, or immune escape mechanisms, which have been increasingly recognized in patients receiving immunotherapy. These findings highlight the limitations of relying solely on primary tumor response to evaluate overall disease control (24–27).

In the present case, the diagnostic complexity was further increased by the use of immune checkpoint inhibitors (ICIs). With the expanding application of immunotherapy, immune-related adverse events (irAEs) have become an important consideration in patients presenting with new-onset symptoms (28–30). ICIs can induce a spectrum of genitourinary irAEs, including immune-related cystitis, ureteritis, and acute interstitial nephritis (AIN), which may present with dysuria, frequency, urgency, hematuria, and radiologic findings such as bladder wall thickening and hydronephrosis (6, 31–33). These features are non-specific and may closely mimic metastatic bladder involvement, creating a critical diagnostic pitfall: misattribution to irAEs could inappropriately delay oncologic management (24, 34). Therefore, this case underscores the necessity of early pathological confirmation (6, 34). Specifically, when a patient on PD-1 plus chemotherapy develops new lower urinary tract symptoms or bladder wall thickening, clinicians should not default to an irAE explanation without performing tissue sampling. Timely biopsy is critical to exclude malignant progression, as an accurate diagnosis governs subsequent therapeutic decisions regarding immunotherapy continuation or salvage therapy conversion.

However, multiple clinical and endoscopic features in this patient argued against an isolated immune-related toxicity. First, imaging demonstrated diffuse and irregular bladder wall thickening with progressive bilateral hydronephrosis. Second, cystoscopy revealed diffuse follicular mucosal lesions with obstruction of both ureteric orifices, whereas immune-related cystitis more commonly presents with non-specific mucosal erythema and edema. Bladder biopsy confirmed poorly differentiated adenocarcinoma with signet-ring cell components. Immunohistochemical staining for CK20, CDX2, and Villin was positive, supporting gastric origin. Attributing these findings solely to irAEs may delay definitive diagnosis and compromise timely and standardized oncological management.

Another key clinical issue illustrated by this case is the development of acute post-renal obstruction, which represents a urological emergency requiring prompt intervention (35). Although percutaneous nephrostomy effectively relieved obstruction and temporarily improved renal function, it had a limited effect on the overall disease course. This observation is consistent with previous reports indicating that local decompressive procedures, while essential for symptom control and organ preservation, do not alter the natural course of advanced malignancies, particularly in the context of highly aggressive tumor biology (36, 37).

Therefore, in patients receiving immunotherapy who develop new urinary symptoms or atypical imaging findings, clinicians should remain vigilant for genitourinary irAEs while actively excluding metastatic progression, particularly in individuals with aggressive histological subtypes, such as signet-ring cell carcinoma. Early cystoscopy with targeted biopsy is essential for establishing an accurate diagnosis and guiding rational treatment.

## Conclusion

Bladder metastasis should be considered in patients with gastric cancer who develop novel urinary symptoms during immunotherapy. Early pathological confirmation is essential for ensuring accurate diagnosis and appropriate management. Even in patients who initially respond to treatment, rapid disease progression may occur, reflecting the aggressive nature of the disease.

## Data Availability

The original contributions presented in the study are included in the article/supplementary material. Further inquiries can be directed to the corresponding author.
